# The Pharmacological Management of Ketamine Use Disorder: A Systematic Review

**DOI:** 10.1097/ADM.0000000000001340

**Published:** 2024-06-26

**Authors:** Emmert Roberts, Elizabeth Sanderson, Irene Guerrini

**Affiliations:** From the National Addiction Centre, Institute of Psychiatry, Psychology and Neuroscience (IoPPN), King’s College London, United Kingdom and South London, and the Maudsley NHS Foundation Trust, United Kingdom (ER); and South London and the Maudsley NHS Foundation Trust, United Kingdom (ES, IG).

**Keywords:** dependence, ketamine, intervention, pharmacotherapy, systemic review

## Abstract

**Objectives:**

There has been limited evidence synthesis examining treatment of ketamine use disorder. We aimed to conduct a systematic review to assess the efficacy and tolerability of pharmacological interventions in the management of ketamine use disorder.

**Methods:**

We searched MEDLINE, EMBASE, PsychINFO, and CENTRAL (Cochrane Central Register of Controlled Trials) from database inception to November 14, 2023, for studies of any design that reported on any pharmacological intervention in the management of ketamine use disorder. We extracted any reported measure of efficacy or tolerability and assessed outcome quality using the GRADE (Grading of Recommendations Assessment, Development, and Evaluation) framework. We planned to combine outcomes using random-effects meta-analysis, where this was not possible results were reported narratively.

**Results:**

Twelve studies met the inclusion criteria reporting on 368 participants. These comprised 1 controlled trial, 2 retrospective case series, and 9 case reports. Two studies reported on ketamine intoxication, 6 on withdrawal, and 4 on craving/relapse prevention. All studies reported only descriptive outcomes, and all evidence was of very low quality. Benzodiazepine regimens and haloperidol were reported to have potential utility in intoxication and withdrawal, whereas naltrexone, lamotrigine, and a combination of paliperidone palmitate and bupropion were reported to have potential utility in craving/relapse prevention.

**Conclusions:**

There is a paucity of research into pharmacological management of ketamine use disorder. The limited very low-quality evidence suggests benzodiazepine regimens may be most salient for future exploration in management of ketamine intoxication and withdrawal, whereas case reports suggest naltrexone, lamotrigine, and paliperidone palmitate plus bupropion may potentially merit further investigation with regard to craving/relapse prevention.

Ketamine has been widely used in human and veterinary medicine for over 40 years, exerting its primarily dissociative effects through noncompetitive antagonism at the *N*-methyl-d-aspartate receptor.^1^ There have been substantial recent expansions in its therapeutic use with emerging effectiveness in a variety of mental and behavioral disorders including treatment-resistant depression.^2^ Recreational use and dependence have been consistently described with a number of reports over the past decade highlighting a concerning increase in the prevalence of ketamine use disorder.^3,4^ In 2019, in the United States, self-reported past-year nonmedical use was reported to reach a peak prevalence of 0.9%.^5^

Despite these concerns, comparatively little research has been conducted into treatment strategies for ketamine use disorder as opposed to its use as a therapeutic agent.^1^ Indeed, recent work has focused on the potential of ketamine in the management of addiction disorders without a strong focus on the management of ketamine addiction itself.^6^ Although systematic reviews of interventions for complications of ketamine use disorder, for example, ketamine-related uropathy,^7^ have been conducted to our knowledge, no analogous evidence syntheses examining the pharmacological management of ketamine use disorder are available in the literature.

As ketamine use becomes more widespread in psychiatric practice, increased consumption has the potential to result in more widespread cases of extramedical use, and health care professionals may have a greater need to understand both the current evidence base of treatment options and potential worthwhile avenues of future research.^8^ To address this gap, we aimed to conduct a systematic review to assess the efficacy and tolerability of pharmacological interventions in the management of ketamine use disorder. Additionally, we planned to use the GRADE (Grading of Recommendations Assessment, Development, and Evaluation) methodology to assess the quality of the evidence and the strength of any resultant recommendations.

## METHODS

This study is reported according to the PRISMA (Preferred Reporting Items for Systematic Reviews and Meta-analyses) guidelines,^9^ with the review protocol preregistered on PROSPERO (registration number: CRD42023483043).

### Search Strategy

We searched MEDLINE, EMBASE, PsychINFO, and the Cochrane Central Register of Controlled Trials (CENTRAL) from database inception to November 14, 2023. The complete search strategy can be found as Figure S1 the online supplementary material, http://links.lww.com/JAM/A526.

### Study Selection

Two authors (ER and ES) initially assessed titles and abstracts and reviewed the full text of the remaining articles for inclusion. Any discrepancy was resolved by discussion, and where agreement could not be reached a third author (IG) was consulted. All relevant references were checked for additional citations.

The review protocol can be found as Table S1 in the online supplementary material, http://links.lww.com/JAM/A526. We included studies of any design where any pharmacological intervention had been used to manage a mental or behavioral aspect of problematic ketamine use or ketamine use disorder. This included treatment of ketamine intoxication, withdrawal, harmful use, dependence, craving, and/or relapse prevention. We excluded studies where pharmacological interventions were specifically targeting any comorbid physical, mental, or behavioral disorder in patients with ketamine use disorder. Full descriptions of each study’s included population, intervention/s, and, where applicable, comparison/s can be found in Table 1.

**TABLE 1:** Included Studies

	Study ID	Study Design	Population					Intervention	Comparison	Outcomes
			Description	n	Female:Male	Age, y	Country			
Intoxication	Giannini et al, 2000^10^	Controlled trial	“Patients found to have abused ketamine and no other substance”*	21	0:21	22–30	USA	5 mg intramuscular haloperidol (0, 30, and 60 min)	Placebo	Significantly lower Brief Psychiatric Rating Scale total scores in the intervention group at 30 and 60 min
	Ng et al, 2010^11^	Retrospective case series	“Presentation to 1 of 15 emergency departments following ketamine use within 48 hours, or a urine test positive for ketamine”	233	74:159	Median, 22 Range, 13–60	Hong Kong	“Benzodiazepines for agitation”	N/A	“Most of the patients (197/233, 85%) developed no or only minor complaints; the majority (168/233, 72%) were safely managed in the emergency department”
Withdrawal	Pal et al, 2002^12^	Case report	“Injection use of… ketamine for 5 to 6 years admitted to the in-patient unit”	1	0:1	28	India	“Diazepam over the first few days”	N/A	“Mild discomfort which settled”
	Lim, 2003^13^	Case reports	“Ketamine dependent patient… elected for inpatient withdrawal”	1	0:1	“Mid 30s”	Singapore	“Two days of lorazepam 3 mg/day, propranolol 40 mg/day, diazepam 10 mg Nocte and naltrexone 50 mg/day”	N/A	“Psychotic symptoms resolved quickly”
			“Ketamine dependent patient… seeking voluntary detoxification from a general hospital”	1	0:1	“Early 30s”	Singapore	“A short course of haloperidol up to 4.5 mg/day”	N/A	“The acute psychotic episode lasted 6 days and resolved”
	Critchlow, 2006^14^	Case report	“A six-year history of ketamine misuse… [who had] decided to undergo detoxification”	1	0:1	25	United Kingdom	“A reducing dose of diazepam from 20 mg over 3 days”	N/A	“The regimen was successful and eliminated the majority of withdrawal symptoms”
	Blachut et al, 2009^15^	Case report	“Addicted to ketamine for 15 years… [displaying] a withdrawal syndrome as an effect of abrupt discontinuation”	1	0:1	52	Poland	“Diazepam, carbamazepine, and vitamins were used during treatment”	N/A	“The patient was then motivated to stop using ketamine”
	Chen et al, 2020^16^	Retrospective case series	“Receiving inpatient treatment for ketamine withdrawal”	104	28:76	Mean, 30.3 SD, 6.1	Taiwan	“5 mg Diazepam administered orally 4 times a day, starting from the first day of admission, then gradually tapered off before day 5 and only given for as-needed use afterward”	N/A	“Patients who had higher baseline Beck Depression Inventory scores, more cravings, and more frequent ketamine use were more likely to require longer treatment for withdrawal”
	Roxas et al, 2021^17^	Case report	“Escalating 8 months of monthly intravenous ketamine infusions and 100-mg oral lozenges taken 4 times daily—involuntarily admitted to an inpatient unit”	1	0:1	35	USA	“10 mg olanzapine once at night, 2 mg lorazepam 3 times/day, and 0.1 mg clonidine twice a day for 2 days”	N/A	“He was calmer, more cooperative, and less labile, although he still reported racing thoughts”
Anticraving/relapse prevention	Garg et al, 2014^18^	Case report	“Injecting up to 4 g of ketamine per day”	1	0:1	“Early 40s”	India	“Naltrexone in doses of 25 mg/day escalated to 50 mg/day”	N/A	“Craving subsided with agreement to stay in the treatment… Maintained complete abstinence from ketamine for 12 months on naltrexone”
	Perez Gomez et al, 2015^19^	Case report	“Serious ketamine addiction up to 5–6 g/day”	1	0:1	38	Spain	“Paliperidone palmitate in increasing doses from 75 to 150 mg combined with bupropion in high doses”	N/A	“From the start of treatment the patient is abstinent of ketamine. Impulsivity and dysphoria have improved and suicide ideation has gone”
	Bhad et al, 2016^20^	Case report	“Injectable ketamine use by intramuscular route for 4 years”	1	0:1	48	India	“Naltrexone 50 mg/day as an anticraving agent”	N/A	“Ketamine dependence was managed with naltrexone”
	Huang et al, 2016^21^	Case report	“Inability to discontinue ketamine use for at least 1 year despite marked functional impairment and severe physical consequences”	1	0:1	25	Taiwan	“Slow titration of lamotrigine to 100 mg/day”	N/A	“Markedly decreased use frequency and decreased daily use… Craving scores in visual analog scale decreased from 10 to 3 after taking lamotrigine”

*This study was originally intended to be conducted in patients with phencyclidine intoxication, but ketamine was discovered to have been the sole agent present in this subset of the participants based on serum and urine results.

### Data Extraction

Two authors (ER and ES) independently extracted data from all eligible studies using a standardized data extraction spreadsheet, which can be found as Table S2 in the online supplementary material, http://links.lww.com/JAM/A526. In the case of incomplete reporting of data, we searched studies’ online supplementary appendices and contacted authors as necessary. The main outcomes extracted were any reported measure or description of efficacy or tolerability.

### Quality Assessment

Where possible, outcome quality was assessed using the GRADE framework with risk of bias in any included randomized trials assessed using the Cochrane Risk of Bias Assessment Tool 2 (RoB 2),^22^ and in observational studies using the applicable adapted Newcastle Ottawa Scale.^23^ Two reviewers (ER and ES) independently scored the quality of each study. Any discrepancy was resolved by discussion, and where agreement could not be reached, a third author (IG) was consulted. A complete description of the GRADE and Newcastle Ottawa Scale quality scoring can be found as Tables S4 and S5 in the online supplementary material, http://links.lww.com/JAM/A526.

### Statistical Analysis

Where sufficient data were presented, we had planned to combine outcomes in pairwise meta-analysis; however, this was ultimately not possible because of lack of available data. As high levels of heterogeneity were anticipated, we planned to use a random-effects model. All analyses were conducted using Stata IC version 17 (StataCorp, College Station, Tex), the significance level set at 0.05. Where only descriptions of efficacy and/or tolerability outcomes were stated, these were reported narratively.

## RESULTS

The search generated 2067 unique results, and 7 additional references were identified from citation searching leading to a total of 2074. We examined 72 full texts and included 12 studies. Sixty studies were excluded, common reasons for exclusion being that studies did not report use of a pharmacological intervention in ketamine use disorder or ketamine itself was being used as an intervention to treat another health condition. Full reasons for each study’s exclusion can be found as Table S3, http://links.lww.com/JAM/A526, in the online supplementary material alongside the PRISMA diagram in Figure S2, http://links.lww.com/JAM/A526, describing study selection.

The 12 included studies comprised 1 controlled trial, 2 retrospective case series, and 9 case reports. Two studies reported on pharmacological treatments for ketamine intoxication, 6 for withdrawal, and 4 for craving or relapse prevention. All studies reported only descriptive outcomes, and as such, meta-analysis was not possible. All results are thus reported narratively, and a summary of all included studies, with outcomes described verbatim, is available in Table 1.

### Intoxication

One controlled trial with 21 male participants compared three 5 mg intramuscular injections of haloperidol to placebo and reported “significantly lower scores on the Brief Psychiatric Rating Scale,” a clinician-rated instrument measuring 18 behavioral symptoms on an 8-point (0–7) scale, in the haloperidol arm at 30 and 60 minutes.^10^

One retrospective case series in 233 participants described the use of “benzodiazepines for agitation” and reported that “most of the patients (197/233, 85%) developed no or only minor complaints… [with] the majority (168/233, 72%) safely managed in the emergency department.” The study, however, did not specify the number of participants who received benzodiazepines and noted agitation/irritability as a presenting symptom in only 10 cases.^11^

There were serious methodological concerns with the controlled trial, resulting in a very low-quality GRADE assessment. All other available evidence in ketamine intoxication treatment was of very low quality.

### Withdrawal

Five case reports, describing 6 male participants, and 1 retrospective case series in 104 participants reported on different pharmacological strategies to treat ketamine withdrawal.

Three studies described regimens using only oral diazepam. The retrospective case series was conducted in a voluntary ward-based abstinence treatment program (including both pharmacological and nonpharmacological management), where discharge was allowed after a week or when withdrawal symptoms were fully remitted.^16^ The study described “5 mg diazepam… 4 times a day, gradually tapered off before day 5 and given for as-needed use afterward” and reported that “Patients who had higher baseline Beck Depression Inventory scores, more cravings, and more frequent ketamine use were more likely to require longer treatment for withdrawal.” One case report of a patient voluntarily undergoing detoxification and motivational treatment described “a reducing dose of diazepam from 20 mg over 3 days” and reported that “The regimen was successful and eliminated the majority of withdrawal symptoms.”^14^ The final case report described a man voluntarily admitted to an inpatient unit treated with “diazepam over the first few days” having led to “settling of mild discomfort.”^12^

Three case reports described regimens using oral benzodiazepines with adjunctive agents. One case report described an elective inpatient detoxification using “Two days of lorazepam 3 mg/day, propranolol 40 mg/day, diazepam 10 mg Nocte and naltrexone 50 mg/day” and reported that “Psychotic symptoms resolved quickly.”^13^ One case report described a patient admitted to hospital following abrupt ketamine discontinuation treated with a regimen of “diazepam, carbamazepine, and vitamins” that resulted in the patient being “motivated to stop using ketamine.”^15^ The final case report described a patient involuntarily admitted to an inpatient unit treated with “10 mg olanzapine once at night, 2 mg lorazepam 3 times/day, and 0.1 mg clonidine twice a day for 2 days”; this led to the patient being “calmer, more cooperative, and less labile, although he still reported racing thoughts.”^17^

One case report described a patient admitted following arrest and treated with “a short course of haloperidol up to 4.5 mg/day” and that “the acute psychotic episode lasted 6 days and resolved.”^13^

All available evidence in ketamine withdrawal treatment was of very low quality.

### Relapse Prevention

Four case reports, describing 4 male participants, reported on 3 unique pharmacological interventions to promote relapse prevention or the treatment of craving in the context of successful withdrawal.

Two case reports described the use of oral naltrexone at 50 mg/day, with one reporting that the patient’s “Craving subsided… [and he] maintained complete abstinence from ketamine for 12 months on naltrexone.”^18^ The second report simply stated the case was “managed with naltrexone.”^20^

One case described the use of oral lamotrigine at 100 mg/day reporting that the patient’s ketamine use “Markedly decreased… [and] craving scores in visual analog scale decreased from 10 to 3 after taking lamotrigine.”^21^

A final case described the use of 150 mg intramuscular paliperidone palmitate combined with “bupropion in high doses” and reported that “From the start of treatment, the patient is abstinent of ketamine. Impulsivity and dysphoria have improved, and suicide ideation has gone.”^19^

All available evidence in treatment of craving or relapse prevention was of very low quality.

### Tolerability

No studies reported on any safety outcomes, nor did they describe any concerns with regard to pharmacological intervention tolerability.

## DISCUSSION

Only very low-quality evidence of efficacy and tolerability was available for any pharmacological intervention in the treatment of ketamine use disorder. There were no randomized trials, and apart from one small controlled study with serious methodological concerns, all published articles were uncontrolled, with the majority of evidence confined to case reports. Both benzodiazepine regimens and haloperidol were reported to have utility in the management of ketamine intoxication and withdrawal, the former as both a monotherapy and in combination with several adjuncts, and the latter particularly in stabilization of acute psychotic symptoms, whereas in the management of craving or relapse prevention, case-report-level evidence described the potential utility of naltrexone, lamotrigine, and a combination of paliperidone palmitate and bupropion. No published studies reported on any safety outcomes, nor did they describe any concerns with regard to pharmacological intervention tolerability.

The study has several strengths and limitations. We implemented robust methods to conduct the review, using a broad search strategy and a predefined protocol to capture evidence from studies of any design. Three-quarters of published studies were case reports, and as such, meta-analysis could not be performed. GRADE scoring was appropriate for only a single outcome, and evidence for all reported outcomes deemed very low quality. Although the review only examined the evidence base for pharmacological interventions and did not assess the efficacy or tolerability of psychological treatments, recent guidelines acknowledge limited research in this area with no current recommended psychological treatment options.^4^ Of the overall 368 included participants, only 102 (27.7%) were female, with all case reports describing male subjects, highlighting the need to redress this inequity when conducting future research.

There is an overall paucity of research with no randomized evidence available despite a substantial and increasing burden of ketamine use disorder globally; as such, well-designed placebo-controlled randomized controlled trials within this area should be prioritized for future research.^8,24^ People with underlying mental illness have consistently been shown to be more susceptible to development of comorbid addiction disorders,^25^ and as ketamine prescription becomes more commonplace in this population, professionals should be aware of the limited evidence of effective treatments prior to considering ketamine prescription.^4^ Future research should ensure methodological rigor, use validated scales to assess symptomatology, and aim for equity with regard to gender representation.

## CONCLUSIONS

When considering possible intervention, patients and professionals need to understand that the current evidence base available for pharmacological treatment in ketamine use disorder is limited and of very low quality. Although this review demonstrates a clear need to investigate novel compounds in the management of ketamine use disorder, there are some promising avenues for future potential research based on the published literature. Given their common use in other addiction disorders and the limited very low-quality evidence available, benzodiazepine regimens would appear the most salient treatment option for future exploration in management of ketamine intoxication and withdrawal; with case-report-level evidence the only empirical data available, these suggest that naltrexone, lamotrigine, and paliperidone palmitate plus bupropion may potentially merit further investigation with regard to craving and relapse prevention.
